# Genome sequence of *Acuticoccus yangtzensis* JL1095T (DSM 28604T) isolated from the Yangtze Estuary

**DOI:** 10.1186/s40793-017-0295-6

**Published:** 2017-12-29

**Authors:** Lei Hou, Jia Sun, Xiabing Xie, Nianzhi Jiao, Yao Zhang

**Affiliations:** 1 State Key Laboratory of Marine Environmental Sciences, Xiamen University, Xiamen, 361102 People’s Republic of China; 2 Institute of Marine Microbes and Ecospheres, Xiamen University, Xiamen, 361102 People’s Republic of China

**Keywords:** *Acuticoccus yangtzensis* JL1095^T^, Aromatic compounds degradation, Methane metabolism, Form II CODH, Aerobic CO oxidation, Yangtze estuary

## Abstract

*Acuticoccus yangtzensis* JL1095^T^ is a proteobacterium from a genus belonging to the family *Rhodobacteraceae*; it was isolated from surface waters of the Yangtze Estuary, China. This strain displays the capability to utilize aromatic and simple carbon compounds. Here, we present the genome sequence, annotations, and features of *A. yangtzensis* JL1095^T^. This strain has a genome size of 5,043,263 bp with a G + C content of 68.63%. The genome contains 4286 protein-coding genes, 56 RNA genes, and 83 pseudo genes. Many of the protein-coding genes were predicted to encode proteins involved in carbon metabolism pathways, such as aromatic degradation and methane metabolism. Notably, a total of 31 genes were predicted to encode form II carbon monoxide dehydrogenases, suggesting potential for carbon monoxide oxidation. The genome analysis helps better understand the major carbon metabolic pathways of this strain and its role in carbon cycling in coastal marine ecosystems.

**Electronic supplementary material:**

The online version of this article (10.1186/s40793-017-0295-6) contains supplementary material, which is available to authorized users.

## Introduction

We isolated a member in the family 10.1601/nm.1037, 10.1601/nm.26592 JL1095^T^ (= 10.1601/strainfinder?urlappend=%3Fid%3DCGMCC+1.12795 = 10.1601/strainfinder?urlappend=%3Fid%3DDSM+28604), from surface waters of the Yangtze Estuary, China (31° N, 122° E) [1, 2]. The physiological properties of members in the family 10.1601/nm.1037 suggest that they may be important in regulating the carbon cycle in terrestrial and marine ecosystems. For instance, many members of this family can degrade aromatic compounds [3] and metabolize one-carbon compounds [4]. Physiological tests of 10.1601/nm.26592 JL1095^T^ have shown that strain JL1095^T^ was able to degrade naphthol-AS-BI-phosphate, and utilize acetic acid and glycerol [1]. In addition, many members of the family 10.1601/nm.1037 examined to date have the ability to oxidize CO.

CO is an important atmospheric trace gas that contributes to climate change despite its low concentrations (0.05–0.12 ppm) in air [5]. Although CO is toxic for many organisms, a number of microbes can consume CO. Marine microbial CO oxidation represents an important CO sink in the oceans. CODHs, key enzymes for CO oxidation, have been classified into two major types based on their cofactor composition, structure, and stability in the presence of dioxygen [6]. Ni- and Fe-containing CODHs are found in anaerobic bacteria and archaea, while Cu- and Mo-containing CODHs are found in aerobic bacteria [7]. Compared with the relatively hypoxic and high CO concentrations in the early Earth environment [8], the ecological significance of aerobic CO oxidation has become increasingly critical in the relatively aerobic and low CO concentrations in modern environments. Aerobic CO oxidation is carried out by phylogenetically and physiologically diverse aerobic bacteria and certain newly identified archaea that are distributed in a variety of habitats, including terrestrial, sedimentary, freshwater, and marine ecosystems [9]. The most active CO oxidizers belong to various genera, such as 10.1601/nm.1144, 10.1601/nm.1134, 10.1601/nm.1155 and 10.1601/nm.1150, mostly from the family 10.1601/nm.1037 [10, 11]. Based on phylogenic analysis of 16S rRNA sequences and physiological characteristics, 10.1601/nm.26592 JL1095^T^ is most closely related to the genus 10.1601/nm.1155 [1], in which all known and examined to date have the ability to oxidize CO, containing form I and II *cox* gene operons [12–14].

In this study, we describe the classification and features of 10.1601/nm.26592 JL1095^T^, report its first draft genome sequence, and explore its major carbon metabolic pathways and potential capability to oxidize CO.

## Organism information

### Classification and features

**Fig. 1:**
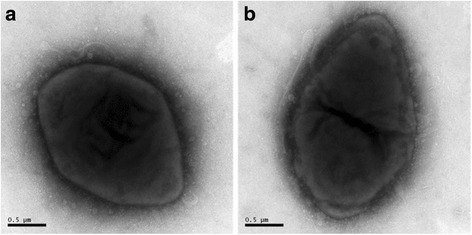
Transmission electron micrographs of 10.1601/nm.26592 JL1095^T^ cultured on marine agar 2216 (MA; Difco) medium. **a** Oval-shaped cells with one peak end; **b** a cell divided by binary fission. Scale bar, 0.5 μm

10.1601/nm.26592 JL1095^T^ (= 10.1601/strainfinder?urlappend=%3Fid%3DCGMCC+1.12795 = 10.1601/strainfinder?urlappend=%3Fid%3DDSM+28604), as the type strain of 10.1601/nm.26592 in the family 10.1601/nm.1037, is a Gram-negative, aerobic, motile (possibly through gliding), oval-shaped with one peak end bacterium (Fig. 1). The detailed classification and features were previously reported [1, 2]. Briefly, the solo-carbon-source utilization test indicated that Tween 40, Tween 80, L-arabinose, methyl-pyruvate, *β*-hydroxy butyric acid, D,L-lactic acid, acetic acid, urocanic acid, *α*-hydroxy butyric acid, *γ*-hydroxy butyric acid, L-proline, glycerol, *α*-keto butyric acid, D-fructose, L-fucose, D-galactose, *α*-D-glucose, D-mannose, L-serine, D-sorbitol, D-gluconic acid, *α*-keto glutaric acid, succinamic acid, L-glutamic acid, pyruvate, and gelatin were utilized by this strain. In addition, strain JL1095^T^ produces various enzymes for the degradation of organic matter, including urease, protease, alkaline phosphatase enzyme, esterase (C4), leucine arylamidase, valine arylamidase, trypsin and naphthol-AS-BI-phosphate hydrolase [1]. The current classification and general features of 10.1601/nm.26592 JL1095^T^ are listed in Table 1.

**Table 1:** Classification and general features of 10.1601/nm.26592 strain JL1095^T^ [16]

MIGS ID	Property	Term	Evidence code^a^
	Classification	Domain *Bacteria*	TAS [30]
		Phylum 10.1601/nm.808	TAS [31]
		Class 10.1601/nm.809	TAS [32]
		Order 10.1601/nm.1036	TAS [33]
		Family 10.1601/nm.1037	TAS [33]
		Genus 10.1601/nm.26591	TAS [1, 2]
		Species 10.1601/nm.26592	TAS [1, 2]
		Type strain: *JL1095* ^*T*^ *(=* 10.1601/strainfinder?urlappend=%3Fid%3DCGMCC+1.12795 *=* 10.1601/strainfinder?urlappend=%3Fid%3DDSM+28604 *)*	
	Gram stain	Negative	TAS [1]
	Cell shape	Oval-shaped with one peak end	TAS [1]
	Motility	Motile	TAS [1]
	Sporulation	Not reported	NAS
	Temperature range	15–50 °C	TAS [1]
	Optimum temperature	35 °C	TAS [1]
	pH range; Optimum	6.0–9.0; 7.6	TAS [1]
	Carbon source	Tween 40, Tween 80, _L_-arabinose, methyl-pyruvate, _D,L_-Lactic acid, acetic acid, urocanic acid, α-hydroxy butyric acid, β-hydroxy butyric acid and γ-hydroxy butyric acid	TAS [1]
MIGS-6	Habitat	Estuary	TAS [1]
MIGS-6.3	Salinity	2–10% NaCl (*w*/*v*)	TAS [1]
MIGS-22	Oxygen requirement	Aerobic	TAS [1]
MIGS-15	Biotic relationship	free-living	NAS
MIGS-14	Pathogenicity	Non-pathogen	NAS
MIGS-4	Geographic location	Yangtze Estuary, China	TAS [1]
MIGS-5	Sample collection	January 2006	IDA
MIGS-4.1	Latitude	31° N	TAS [1]
MIGS-4.2	Longitude	122° E	TAS [1]
MIGS-4.4	Altitude	Sea level	TAS [1]

^a^Evidence codes - *IDA* Inferred from Direct Assay, *TAS* Traceable Author Statement (i.e., a direct report exists in the literature), *NAS* Non-traceable Author Statement (i.e., not directly observed for the living, isolated sample, but based on a generally accepted property for the species, or anecdotal evidence). These evidence codes are from the Gene Ontology project [22]

**Fig. 2:**
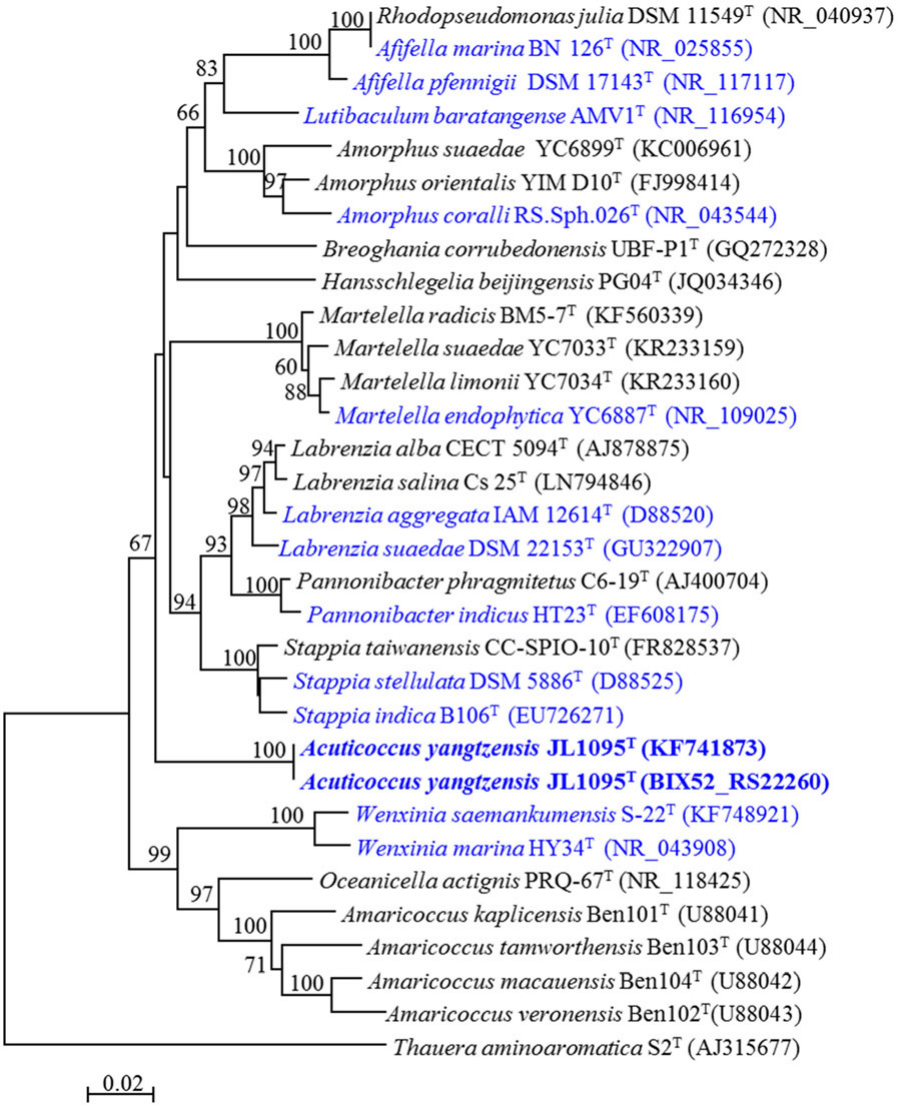
Phylogenetic tree illustrating the relationship between 10.1601/nm.26592 JL1095^T^ and other validly published species. The tree was constructed with MEGA 5.05 software by using the neighbor-joining (NJ) method for 16S rRNA gene sequences. Accession numbers in the GenBank database are shown in parentheses. Reference sequences from relative strains that has been sequenced and obtained a public genome are in blue font, while the JL1095^T^ sequence is in blue bold font. The numbers at the nodes indicate bootstrap percentages based on 1000 replicates; only values higher than 50% are shown. Bar, 0.02 substitutions per nucleotide position. 10.1601/nm.2054 S2^T^ was used to root the tree

The draft genome sequence of 10.1601/nm.26592 JL1095^T^ has one full-length 16S rRNA gene sequence (1450 bp; BIX52_RS22260) that was consistent with the partial 16S rRNA gene sequence from the original species description (1397 bp; KF741873) [1]. Strain JL1095^T^ showed the highest 16S rRNA gene sequence similarity with 10.1601/nm.17875 B106^T^ (92.7%) followed by 10.1601/nm.1155 *stellata* 10.1601/strainfinder?urlappend=%3Fid%3DIAM+12621 ^T^ (92.6%) and 10.1601/nm.25296 10.1601/strainfinder?urlappend=%3Fid%3DDSM+22153 ^T^ (92.3%). The phylogenetic tree was constructed to assess the evolutionary relationships between strain JL1095^T^ and other related strains with the MEGA 5.05 software by using a neighbor-joining algorithm with the Jukes-Cantor model. The phylogeny of the strain JL1095^T^ illustrated that one monophyletic branch is formed at the periphery of the evolutionary radiation occupied by the various genera in the family 10.1601/nm.1037 (Fig. 2).

## Genome sequencing information

### Genome project history

This strain was selected for sequencing on the basis of its important evolutionary position, the degradation of aromatic and simple hydrocarbon compounds via metabolism [1], and its potential CO oxidation ability. The sequencing of the 10.1601/nm.26592 JL1095^T^ genome was carried out at Beijing Novogene Bioinformatics Technology Co., Ltd. The genome sequence of 10.1601/nm.26592 JL1095^T^ has been deposited in the GOLD [15] and DDBJ/EMBL/GenBank under accession number MJUX00000000. A summary for the genome sequencing information of 10.1601/nm.26592 JL1095^T^ is listed in Table 2, in compliance with MIGS version 2.0 [16].

**Table 2:** Project information

MIGS ID	Property	Term
MIGS 31	Finishing quality	High-quality draft
MIGS-28	Libraries used	500 bp Paired-end
MIGS 29	Sequencing platforms	Illumina HiSeq 2500
MIGS 31.2	Fold coverage	331X
MIGS 30	Assemblers	SOAPdenovo version 2.04
MIGS 32	Gene calling method	GeneMarkS version 4.17
	Locus Tag	BIX52
	Genbank ID	MJUX00000000
	GenBank Date of Release	December 31th, 2016
	GOLD ID	Gp0206530
	BIOPROJECT	PRJNA343888
MIGS 13	Source Material Identifier	10.1601/strainfinder?urlappend=%3Fid%3DCGMCC+1.12795=10.1601/strainfinder?urlappend=%3Fid%3DDSM+28604
	Project relevance	Environmental, microbes

### Growth conditions and genomic DNA preparation

10.1601/nm.26592 JL1095^T^ (= 10.1601/strainfinder?urlappend=%3Fid%3DCGMCC+1.12795 = 10.1601/strainfinder?urlappend=%3Fid%3DDSM+28604) was cultivated aerobically in MB (Difco) medium. The genomic DNA of strain JL1095^T^ was extracted using the Tguide Bacteria Genomic DNA Kit (OSR-M502, TIANGEN Biotech Co. Ltd., Beijing, China) in accordance with the instruction manual. After this strain was cultivated in MB medium in the shaker at 35 °C for 2–3 days, the total DNA obtained was subjected to quality control by agarose gel electrophoresis and quantified by Qubit 2.0 fluorometer (Life Technologies, MA, USA).

### Genome sequencing and assembly

The genome sequencing of this strain was conducted using Illumina HiSeq 2500 paired-end sequencing technology under the PE 150 strategy. A total filtered read size of 1674 Mbp was obtained. The filtered reads were assembled by SOAPdenovo version 2.04 software and 29 contigs were generated [17, 18]. Gene prediction was performed on the genome assembly using GeneMarkS version 4.17 [19].

### Genome annotation

Functional annotation of the coding sequences was performed by searching various databases (KEGG [20], NR, COG [21], and GO [22]). The rRNA genes of strain JL1095^T^ were predicted using rRNAmmer software [23], tRNA genes were identified using tRNAscan-SE [24], and sRNA were predicted by BLAST searches against the Rfam database [25]. The online CRISPRFinder program was used for CRISPR identification [26].

## Genome properties

The 10.1601/nm.26592 JL1095^T^ genome was composed of 5,043,263 bp with a G + C content of 68.63%. A total of 4286 protein-coding genes were predicted with an average length of 994 bp, occupying 87.01% of the genome. The genome also contained 56 RNA genes and 83 pseudo genes. Detailed genome statistical information is shown in Table 3. COG categories were assigned to 2522 of the protein-coding genes which were classified into 21 functional groups. The most dominant COG categories were “amino acid transport and metabolism” followed by “general function prediction only”, “function unknown”, and “energy production and conversion”. Detailed gene numbers and percentages related with the COG categories are shown in Table 4. In total, 2470 protein-coding genes were assigned to 153 KEGG metabolic pathways, including key genes involved in carbon metabolism processes such as gluconeogenesis, polycyclic aromatic hydrocarbon degradation, and methane metabolism. In addition, based on the GO database, 1992 protein-coding genes were assigned to molecular function, 1394 genes were assigned to cellular components, and 2646 genes were assigned to biological processes.

**Table 3:** Genome statistics

Attribute	Value	% of Total
Genome size (bp)	5,043,263	100.00
DNA coding (bp)	4,388,143	87.01
DNA G + C (bp)	3,461,191	68.63
DNA scaffolds	28	100.00
Total genes	4425	100.00
Protein coding genes	4286	96.86
RNA genes	56	1.27
Pseudo genes	83	1.88
Genes in internal clusters	NA	NA
Genes with function prediction	3781	85.45
Genes assigned to COGs	2522	56.99
Genes with Pfam domains	3139	70.94
Genes with signal peptides	348	7.86
Genes with transmembrane helices	1043	23.57
CRISPR repeats	3	0.07

*NA*, no analysis

**Table 4:** Number of genes associated with general COG functional categories

Code	Value	%age	Description
J	162	3.78	Translation, ribosomal structure and biogenesis
A	0	0.00	RNA processing and modification
K	139	3.24	Transcription
L	111	2.59	Replication, recombination and repair
B	3	0.07	Chromatin structure and dynamics
D	19	0.44	Cell cycle control, Cell division, chromosome partitioning
V	20	0.47	Defense mechanisms
T	93	2.17	Signal transduction mechanisms
M	126	2.94	Cell wall/membrane biogenesis
N	30	0.70	Cell motility
U	43	1.00	Intracellular trafficking and secretion
O	111	2.59	Posttranslational modification, protein turnover, chaperones
C	223	5.20	Energy production and conversion
G	198	4.62	Carbohydrate transport and metabolism
E	388	9.05	Amino acid transport and metabolism
F	63	1.47	Nucleotide transport and metabolism
H	122	2.85	Coenzyme transport and metabolism
I	138	3.22	Lipid transport and metabolism
P	187	4.36	Inorganic ion transport and metabolism
Q	109	2.54	Secondary metabolites biosynthesis, transport and catabolism
R	378	8.82	General function prediction only
S	232	5.41	Function unknown
–	1764	41.16	Not in COGs

The total is based on the total number of protein coding genes in the genome

## Insights from the genome sequence

We performed a systematic analysis of the protein-coding genes with functional predictions by BLAST searches against the four databases (KEGG, NR, COG, and GO), with E-value <1e − 5 and minimal alignment length of >40%.

Strain JL1095^T^ was predicted to contain most of the genes central to carbon metabolism, including those related to glycolysis/gluconeogenesis, the tricarboxylic acid cycle, and the pentose phosphate pathway. Furthermore, about 198 genes were assigned to COG categories related to carbohydrate transport and metabolism, including fructose, mannose, and galactose metabolism. These carbohydrate metabolic characteristics are generally coincident with those obtained from a sole-carbon-source utilization experiment [1]. The capacity of this strain to degrade aromatic compounds such as naphthol-AS-BI-phosphate has been identified. Approximately 236 genes were involved in 13 KEGG metabolic pathways related to aromatic compounds degradation, such as polycyclic aromatic hydrocarbon, bisphenol, and naphthalene. Aromatic compounds are important environmental organic pollutants because of their persistence in environments, toxicity, and carcinogenic characteristics [27]. Furthurmore, strain JL1095^T^ was annotated to contain 48 genes related to methane metabolism.

**Fig. 3:**
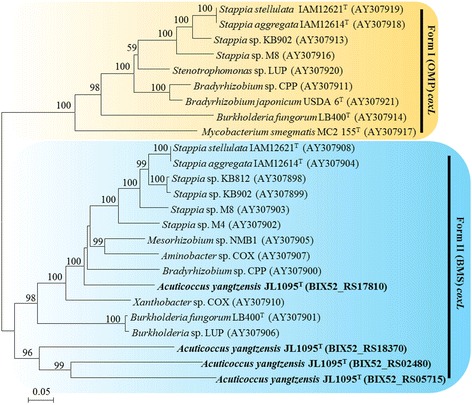
Unrooted phylogenetic tree showing the *coxL* genetype of 10.1601/nm.26592 JL1095^T^. The tree was constructed with MEGA 5.05 software by using the neighbor-joining (NJ) method based on the form I *coxL* and form II putative *coxL* genes from CO-oxidizing microbes. Accession numbers in the GenBank database are shown in parentheses. The *coxL* genes encoded in the 10.1601/nm.26592 JL1095^T^ genome are shown in bold. Sequences in orange and blue shades represent form I and II *coxL* genes, respectively. The numbers at the nodes indicate bootstrap percentages based on 1000 replicates; only values higher than 50% are shown. Bar, 0.05 substitutions per nucleotide position

**Fig. 4:**
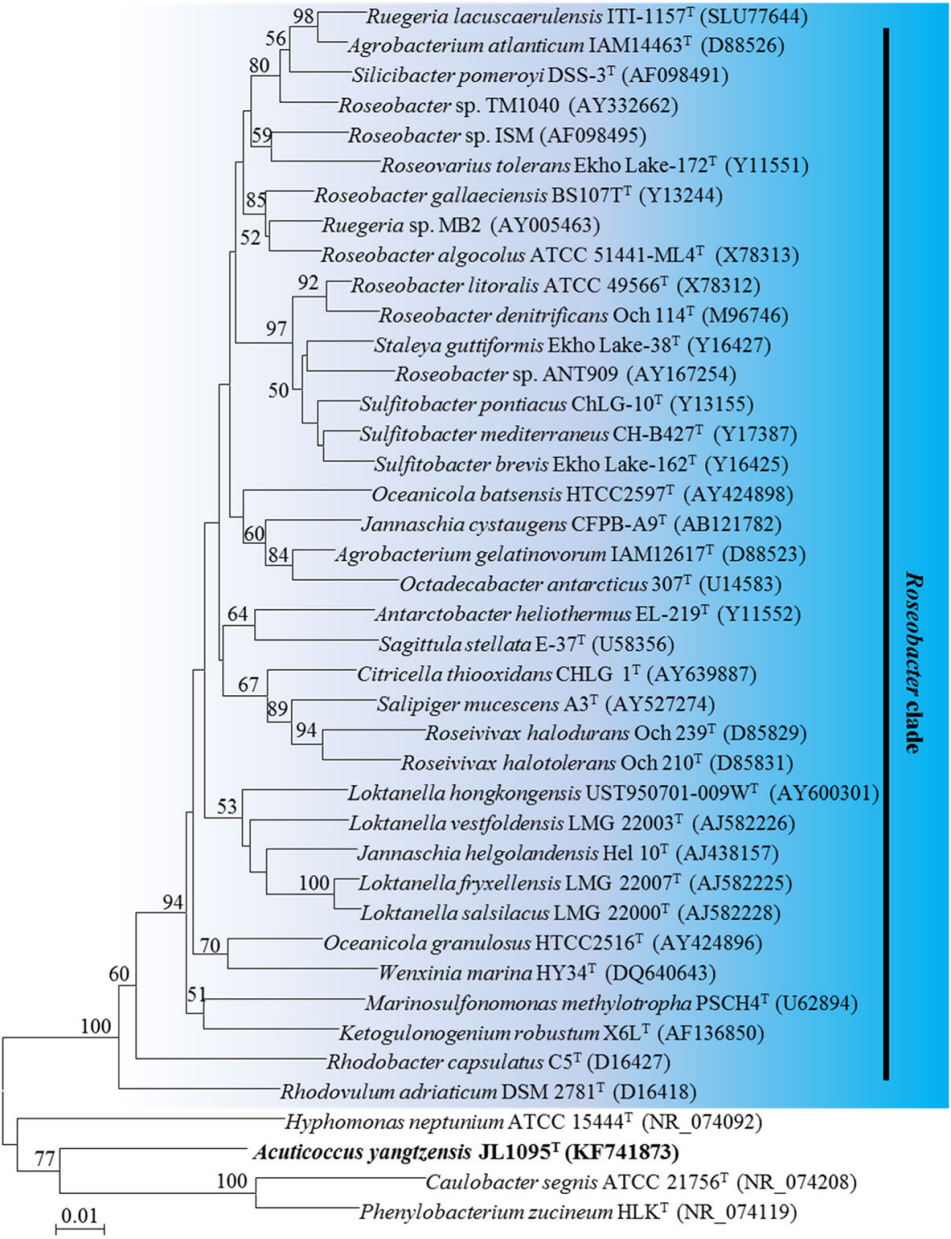
Unrooted phylogenetic tree displaying the relationship between 10.1601/nm.26592 JL1095^T^ and 10.1601/nm.1134 clade bacteria. The tree was constructed with MEGA 5.05 software by using the neighbor-joining (NJ) method based on 16S rRNA gene sequences. Accession numbers in the GenBank database are shown in parentheses. The 16S rRNA gene encoded in the 10.1601/nm.26592 JL1095^T^ genome is shown in bold. The numbers at the nodes indicate bootstrap percentages based on 1000 replicates; only values higher than 50% are shown. Bar, 0.01 substitutions per nucleotide position

Based on results from the four functional annotation databases, the 10.1601/nm.26592 JL1095^T^ genome contained a total of 31 genes predicted to encode aerobic-type CODHs (Additional file 1: Table S1). The *cox* gene clusters that encode aerobic CODHs have been classified into two major forms based on genome analysis [9]. Form I genes are mainly from 10.1601/nm.1489, 10.1601/nm.6310 and 10.1601/nm.2552, and form II putative genes are mainly from 10.1601/nm.1459, 10.1601/nm.1414, and 10.1601/nm.1339 [13]. Form I and II *cox* gene operons consisted of three conserved structural genes that were transcribed as *coxMSL* and *coxSLM*, respectively [28, 29]. For strain JL1095^T^, three structural genes containing *coxS* (small subunit), *coxM* (medium subunit) and *coxL* (large subunit) were all sequenced. Form I *coxS* and *coxM* gene sequences were similar to form II *coxS* and *coxM* gene sequences, but the form II putative *coxL* gene sequence was approximately 40–50% similar to the form I *coxL* gene sequence [9]. Therefore, the *coxL* gene has been used as a molecular biomarker to explore the distribution of aerobic CO bacteria in ecosystems [29]. We constructed the *coxL* phylogenetic tree for strain JL1095^T^ and confirmed that four predicted *coxL* genes (Locus tag: BIX52_RS02480, BIX52_RS05715, BIX52_RS17810 and BIX52_RS18370) were recognized as form II *coxL* genes (Fig. 3). Additionally, the accessory genes were also essential for CO oxidation to take place. The accessory genes in forms I and II varied substantially, and even within the same form, the order and subunit types varied among isolates [9]. Form I *cox* accessory genes, including *coxB*, *C*, *G*, *H*, *I*, and *K*, were distributed flexibly around the structural genes. Among the form II *cox* accessory genes, *coxG* was usually an indispensable gene compared with other accessory genes, such as *coxD*, *E*, and *F* [28]. For this strain, the accessory gene *coxG* was detected. Form I CODH has been specifically characterized for its ability to oxidize CO, while form II is a putative CODH and its ability to oxidize CO remains uncertain. For the 10.1601/nm.1134 clade, both *coxL* forms were present, which enables them to oxidize CO [11]. Phylogenetic analysis using the 16S rRNA gene sequences of 10.1601/nm.26592 JL1095^T^ and 10.1601/nm.1134 clade bacteria indicates that JL1095^T^ does not belong to the 10.1601/nm.1134 clade (Fig. 4). However, many other bacteria containing only form II *cox* genes have been shown by molecular and culture-based methods to oxidize CO, including 10.1601/nm.1414 sp. strain NMB1, 10.1601/nm.1415, 10.1601/nm.1402 sp. strain COX, 10.1601/nm.1573 sp. strain COX, and 10.1601/nm.1619 sp. strain LUP [13]. According to the phylogenetic tree (Fig. 3), the *coxL* genes of JL1095^T^ clustered tightly with these bacterial isolates. Thus, we speculate that JL1095^T^ is capable of oxidizing CO. Future studies are needed to determine its function in CO oxidation.

## Conclusions

In the present study, the genome of 10.1601/nm.26592 JL1095^T^, the type strain of 10.1601/nm.26592, was characterized. It contains numerous genes involved in carbohydrate transport and metabolism, aromatic compounds degradation, and methane metabolism. Knowledge of the genome sequence of 10.1601/nm.26592 JL1095^T^ lays a foundation for better understanding the carbon metabolism of this strain. Based on genome analysis, we speculate that JL1095^T^ is capable of oxidizing CO. Future studies are needed to determine its function in CO oxidation. These genomic data provide insight into the carbon metabolic characteristics of 10.1601/nm.26592 JL1095^T^ and its role in alleviating coastal water pollution and effects on the marine carbon cycle.

## Additional file

Additional file 1: Table S1. Aerobic-type CODH-encoding genes of *Acuticoccus yangtzensis* JL1095^T^ predicted using four different databases. (DOCX 29 kb)

## Abbreviations

CGMCC: China General Microbiological Culture Collection Center
CO: Carbon monoxide
CODHs: CO dehydrogenases
CRISPR: Clustered regularly interspaced short palindromic repeats
DSMZ: Leibniz-Institut DSMZ – Deutsche Sammlung von Mikroorganismen und Zellkulturen GmbH
GOLD: Genomes OnLine Database
MA: marine agar 2216
MB: marine broth 2216
MIGS: Minimum information on the genome sequence
