# Supplementary material for: Genome sequence of Acuticoccus yangtzensis JL1095T (DSM 28604T) isolated from the Yangtze Estuary
Source: Stand Genomic Sci. 2017 Dec 29;12:91. doi: 10.1186/s40793-017-0295-6 (PMC5747140; doi:10.1186/s40793-017-0295-6)
Supplement: Additional file 1: Table S1. — Aerobic-type CODH-encoding genes of Acuticoccus yangtzensis JL1095T predicted using four different databases. (DOCX 29 kb) [file 40793_2017_295_MOESM1_ESM.docx]

**Additional file 1: Table S1**. Aerobic-type CODH-encoding genes of *Acuticoccus yangtzensis* JL1095^T^ predicted using four different databases

| **Locus tag**  BIX52_ | **NR** | **Identity^a^ (%)** | **KEGG** | **Identity^a^ (%)** | **COG** | **Identity^a^ (%)** | **GO** | **Number^b^** |
| --- | --- | --- | --- | --- | --- | --- | --- | --- |
| RS01865 | Xanthine dehydrogenase | 45.42 | *CoxL*/*CutL* | 41.92 | NP | - | NP | - |
| RS01870 | (2Fe-2S)-binding protein | 54.14 | *CoxS*/*CutS* | 50.96 % | *CoxS*/*CutS* | 47.4 % | NP | - |
| RS01875 | hypothetical protein | 48.1 | *CoxM*/*CutM* | 41.18 % | NP | - | oxidoreductase activity | 1 |
| RS02480 | hypothetical protein | 61.43 | *CoxL*/*CutL* | 45.73 % | *CoxL*/*CutL* | 42.4 % | NP | - |
| RS03670 | molybdopterin binding aldehyde oxidase and xanthine dehydrogenase | 69.21 | isoquinoline 1-oxidoreductase, beta subunit | 69.74 % | *CoxL*/*CutL* | 58.98 % | isoquinoline 1-oxido- reductase activity | 2 |
| RS03675 | (2Fe-2S)-binding protein | 72.11 | isoquinoline 1-oxidoreductase, alpha subunit | 70.75 % | *CoxS*/*CutS* | 69.4 % | 2 iron, 2 sulfur cluster binding | 5 |
| RS03815 | *CoxS*/*CutS* | 57.34 | *CoxS*/*CutS* | 53.85 % | *CoxS*/*CutS* | 49.69 % | iron-sulfur cluster binding | 2 |
| RS03820 | hypothetical protein | 45.7 | *CoxM*/*CutM* | 47.2 % | *CoxM*/*CutM* | 41.45 % | NP | - |
| RS03825 | xanthine dehydrogenase, molybdenum binding subunit apoprotein | 63.55 | *CoxL*/*CutL* | 48.77 % | NP | - | NP | - |
| RS05715 | hypothetical protein | 59.68 | *CoxL*/*CutL* | 44.76 % | *CoxL*/*CutL* | 43.18 % | NP | - |
| RS07725 | Periplasmic aromatic aldehyde oxido- reductase, iron-sulfur subunit YagT | 68.47 | xanthine dehydrogenase YagT iron-sulfur-binding subunit | 65.24 % | *CoxS*/*CutS* | 70 % | 2 iron, 2 sulfur cluster binding | 5 |
| RS07730 | hypothetical protein | 76.19 | xanthine dehydrogenase YagS FAD-binding subunit | 74.92 % | *CoxM/CutM* | 74.29 % | flavin adenine dinucleotide binding | 4 |
| RS07735 | molybdenum-binding domain | 65.48 | xanthine dehydrogenase YagR molybdenum-binding subunit | 61.39 % | *CoxL*/*CutL* | 59.15 % | NP | - |
| RS08385 | hypothetical protein | 71.8 | *CoxM*/*CutM* | 59.47 % | *CoxM*/*CutM* | 56.98 % | flavin adenine dinucleotide binding | 4 |
| RS08390 | hypothetical protein | 76.25 | *CoxS*/*CutS* | 73.46 % | *CoxS*/*CutS* | 72.29 % | NP | - |
| RS08395 | carbon monoxide dehydrogenase subunit G | 48.15 | hypothetical protein | 43.55 % | Uncharacterized conserved protein | 55.78 % | NP | - |
| RS12125 | 2Fe-2S subunit | 71.81 | isoquinoline 1-oxidoreductase, alpha subunit | 65.77 % | *CoxS*/*CutS* | 56.46 % | 2 iron, 2 sulfur cluster binding | 5 |
| RS12130 | aldehyde dehydrogenase | 63.61 | isoquinoline 1-oxidoreductase, beta subunit | 57.37 % | *CoxL*/*CutL* | 51.52 % | NP | - |
| RS14355 | xanthine dehydrogenase | 60.59 | ygeT, xdhB xanthine dehydrogenase FAD-binding subunit | 40 % | *CoxM*/*CutM* | 55.68 % | oxidoreductase activity | 1 |
| RS14360 | xanthine dehydrogenase family protein, small/large subunits | 64.88 | NP | - | *CoxL*/*CutL* | 61.73 % | iron-sulfur cluster binding | 2 |
| RS17805 | (2Fe-2S)-binding protein | 75.48 | *CoxS*/*CutS* | 70.12 % | *CoxS*/*CutS* | 70.12 % | iron-sulfur cluster binding | 5 |
| RS17810 | carbon monoxide dehydrogenase | 72.21 | *CoxL*/*CutL* | 45.01 % | *CoxL*/*CutL* | 70.2 % | carbon-monoxide dehydrogenase (acceptor) activity | 3 |
| RS17815 | hypothetical protein | 64.66 | *CoxM*/*CutM* | 59.25 % | *CoxM*/*CutM* | 54.72 % | acting on CH-OH group of donors | 1 |
| RS18370 | hypothetical protein | 64.6 | *CoxL*/*CutL* | 59.11 % | *CoxL*/*CutL* | 56.9 % | oxidoreductase activity | 1 |
| RS20495 | aldehyde oxidase | 80 | NP | - | *CoxL*/*CutL* | 68.39 % | oxidoreductase activity | 2 |
| RS20505 | dehydrogenase | 69.33 | NP | - | *CoxL*/*CutL* | 56.46 % | oxidoreductase activity | 2 |
| RS20510 | ferredoxin | 68.07 | *CoxS*/*CutS* | 51.25 % | *CoxS*/*CutS* | 61.29 % | iron-sulfur cluster binding | 2 |
| RS20515 | oxidoreductase | 50.55 | NP | - | *CoxM*/*CutM* | 48.86 % | NP | - |
| RS20745 | dehydrogenase | 68.33 | xanthine dehydrogenase YagR molybdenum-binding subunit | 66.4 % | *CoxL*/*CutL* | 43.71 % | NP | - |
| RS20750 | molybdopterin dehydrogenase FAD-binding protein | 67.72 | xanthine dehydrogenase YagS FAD-binding subunit | 64.76 % | *CoxM*/*CutM* | 50.15 % | oxidoreductase activity, acting on CH-OH group of donors | 1 |
| RS20755 | (2Fe-2S)-binding protein | 73.12 | xanthine dehydrogenase YagT iron-sulfur-binding subunit | 73.12 % | *CoxS*/*CutS* | 66.89 % | 2 iron, 2 sulfur cluster binding | 6 |

NP, not present; -, no data;

^a^, Identity value (%) of amino acid sequences of JL1095^T^ and three databases (NR, KEGG and COG) sequences alignment;

^b^, The number of genes of JL1095^T^ annotated from the GO database
